# Supplementary figures and images for: 3D-Cultured MC3T3-E1-Derived Exosomes Promote Endothelial Cell Biological Function under the Effect of LIPUS
Source: Biomolecules. 2024 Sep 13;14(9):1154. doi: 10.3390/biom14091154 (PMC11430381; doi:10.3390/biom14091154)

HSP70

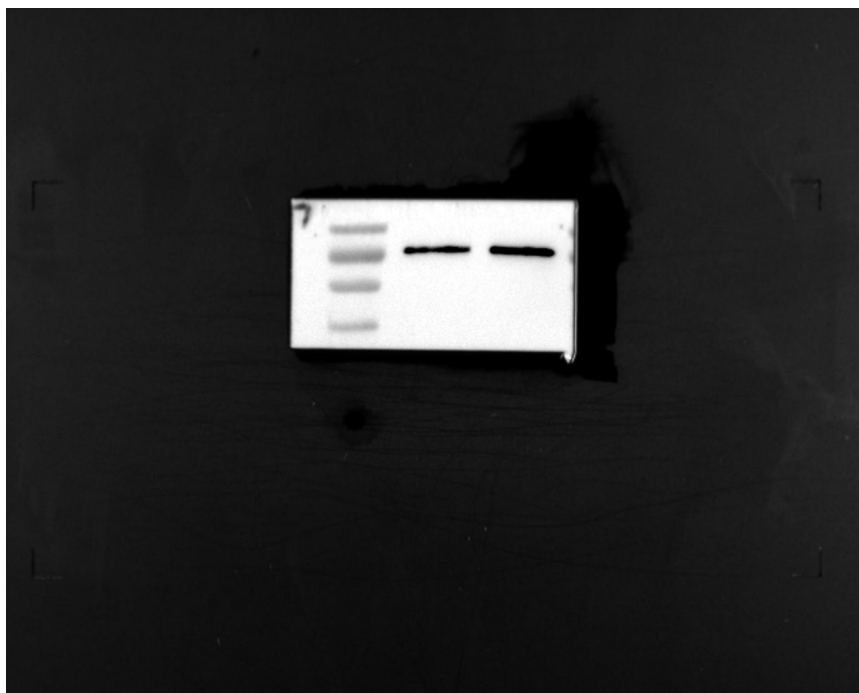

TSG101

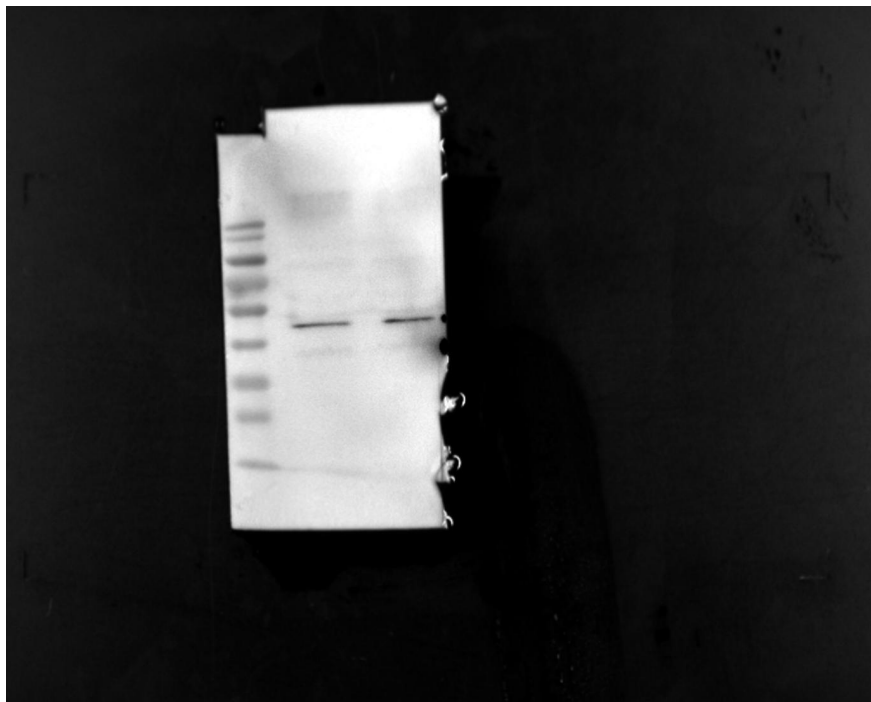

Calnexin

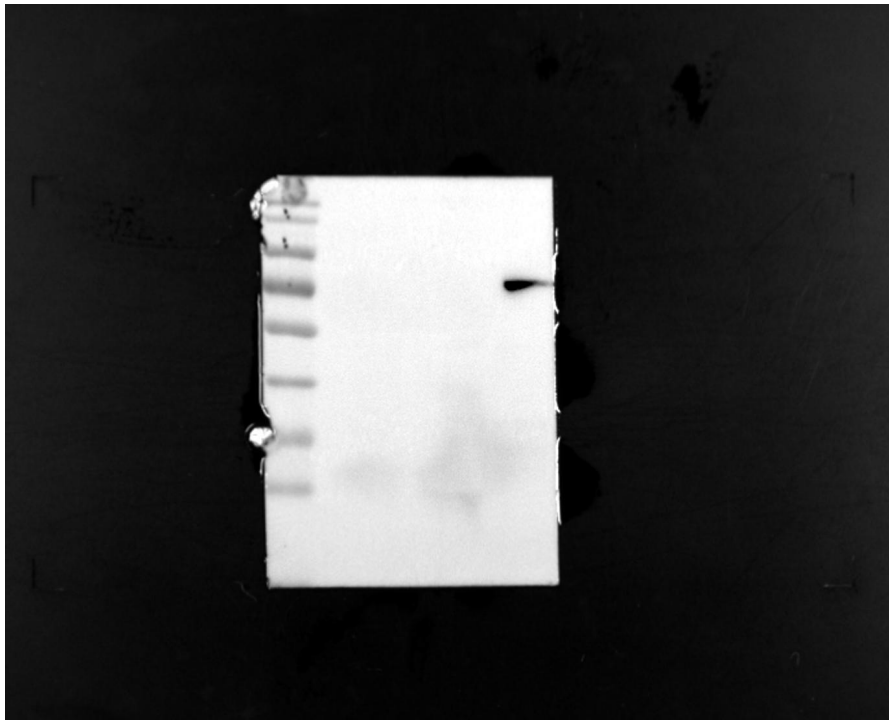

Supplement: Supplementary file 1 [file biomolecules-14-01154-s001.zip › biomolecules-3188735-supplementary.pdf]
